# Gut Microbiota and Depressive Symptoms at the End of CRT for Rectal Cancer: A Cross-Sectional Pilot Study

**DOI:** 10.1155/2021/7967552

**Published:** 2021-12-29

**Authors:** Velda J. Gonzalez-Mercado, Jean Lim, Leorey N. Saligan, Nicole Perez, Carmen Rodriguez, Raul Bernabe, Samia Ozorio, Elsa Pedro, Farrah Sepehri, Brad Aouizerat

**Affiliations:** ^1^Rory Meyers College of Nursing, New York University, New York, NY, USA; ^2^University of Miami, Miami, FL, USA; ^3^Intramural Program, National Institute of Nursing Research/National Institute of Health, Bethesda, MD, USA; ^4^College of Nursing, University of South Florida, Tampa, FL, USA; ^5^Department of General Studies, University of Puerto Rico, San Juan, Puerto Rico; ^6^School of Pharmacy, Medical Science Campus, University of Puerto Rico, San Juan, Puerto Rico; ^7^Bluestone Center for Clinical Research, Department of Oral and Maxillofacial Surgery College of Dentistry, New York University, New York, NY, USA

## Abstract

**Background:**

The role of alterations in gut microbiota composition (termed dysbiosis) has been implicated in the pathobiology of depressive symptoms; however, evidence remains limited. This cross-sectional pilot study is aimed at exploring whether depressive symptom scores changed during neoadjuvant chemotherapy and radiation therapy to treat rectal cancer, and if gut microbial taxa abundances and predicted functional pathways correlate with depressive symptoms at the end of chemotherapy and radiation therapy.

**Methods:**

40 newly diagnosed rectal cancer patients (ages 28-81; 23 males) were assessed for depressive symptoms using the Hamilton Rating Scale for Depression (HAM-D) and provided stool samples for 16S rRNA sequencing. Gut microbiome data were analyzed using QIIME2, and correlations and regression analyses were performed in R.

**Results:**

Participants had significantly higher depressive symptoms at the end as compared to before CRT. The relative abundances of *Gemella*, *Bacillales Family XI*, *Actinomyces*, *Streptococcus*, *Lactococcus*, *Weissella*, and *Leuconostocaceae* were positively correlated (Spearman's rho = 0.42 to 0.32), while *Coprobacter*, *Intestinibacter*, *Intestimonas*, *Lachnospiraceae*, *Phascolarctobacterium*, *Ruminiclostridium*, *Ruminococcaceae (UCG-005 and uncultured)*, *Tyzzerella*, and *Parasutterella* (Spearman's rho = −0.43 to − 0.31) were negatively correlated with HAM-D scores. Of the 14 predicted MetaCyc pathways that correlated with depressive symptom scores at the end of CRT, 11 (79%) were associated with biosynthetic pathways.

**Conclusions:**

Significant bacterial taxa and predicted functional pathways correlated with depressive symptoms at the end of chemotherapy and radiation therapy for rectal cancer which warrants further examination and replication of our findings.

## 1. Introduction

Rectal cancer is prevalent worldwide, with more than 43,000 new patients in the US annually [1]. A large proportion of newly diagnosed rectal cancer patients undergo preoperative neoadjuvant chemotherapy and radiation therapy (CRT) [2, 3]. In addition to the physiological side effects (e.g., diarrhea and constipation), cancer and/or CRT can negatively influence patients' psychological health, precipitating mood disorders, including depression [4, 5]. The prevalence of depression during active treatment (e.g., patients surgically treated) for rectal cancer may be as high as 47% [6], and depression continues to affect 24-31% of patients after treatment completion [4]. However, the literature on the prevalence of depressive symptoms and whether depressive symptom scores changed during CRT for rectal cancer is not available.

Depressive symptoms frequently include loss of interest, sadness, irritability, feelings of worthlessness, hopelessness, guilt or anxiety, concerns over death, or suicidal ideation, as well as associated symptoms such as changes in appetite, weight loss or weight gain, sleep disturbances, psychomotor activity, decreased energy, indecisiveness, or distracted attention [7]. Further, depressive symptoms in cancer patients have been reported to be associated with poor quality of life and lower survival [6, 8]. Unfortunately, depression in patients often goes undiagnosed and undertreated in part because of the lack of laboratory-based diagnostic and prognostic tools [9]. Evidence suggests that the perturbation of the gut microbiota (defined as a diverse collection of microorganisms that inhabit the gastrointestinal tract; [10]) can influence depressive symptoms via the gut-brain axis and that depression phenotyping focusing on the gut microbiome may produce a microbiota-based signature that can serve as a biomarker for depression [11, 12].

Changes in gut microbiota composition induced by factors such as cancer or its treatment have been suggested to be associated or potentially causal of dysbiosis (a disruption in the balance, diversity, and function of symbiotic intestinal microbial communities) [2, 13] in depression and other comorbidities/symptoms [2, 11, 14–16]. We recently reported reduced alpha diversity (Shannon Diversity and Observed Operational Taxonomic Units (OTUs)) in rectal cancer patients in the middle and at the end of CRT compared to before CRT, suggesting that CRT is associated with alterations in gut microbiota composition [15]. However, few microbiome studies had identified specific roles of bacterial taxa in cancer-related comorbidities such as depression. Therefore, this pilot study is aimed at exploring whether depressive symptom scores changed during CRT, and if gut microbial taxa abundances and predicted functional pathways correlate with depressive symptoms at the end of CRT in rectal cancer participants who were not taking antidepressants. Results of this study will further our understanding of the underpinnings of CRT-related depressive symptoms and may inform future strategies for the development of microbial signature/biomarkers to detect intensification of depressive symptoms placing patients at risk for acute depression during CRT in rectal cancer patients.

## 2. Methods

### 2.1. Study Sample and Setting

Prior to data collection, the study protocol received approval by the Institutional Review Board from the Southeastern Academic Medical Center. Patients interested in participating in this study were screened by the research team for eligibility using a brief health history interview that included relevant questions about their cancer history, cancer treatments, current medications, and other health conditions. Forty newly diagnosed rectal cancer patients programmed to receive CRT were included in this study. Eligibility criteria have been described in our previous publications including the following: (1) being at least 18 years of age; (2) no history of inflammatory conditions, infectious conditions, other cancers, sleep disorders, or comorbidities associated with sleep disturbance (e.g., sleep apnea); and (3) with no prior use of antidepressant medications, anti-insomnia medications, antibiotics, probiotics, prebiotics, steroids, or immune-suppressant agents one month before each assessment time-point [2, 14, 15, 17, 18]. Data collection was conducted from September 2017 to April 2019 at three ambulatory radiotherapy facilities in Tampa Bay, Florida, United States. All procedures performed in our study were in accordance both with the ethical standards of the institutional and/or national research committees and with the 1964 Helsinki Declaration and its later amendments or comparable ethical standards. Written informed consents were provided by all participants.

### 2.2. Demographic Characteristics, Clinical Information, and Assessment of Depressive Symptoms

After obtaining informed consent, study participants completed the demographic questionnaire including information on age, gender, and occupation. The research team recorded information from the medical chart including the following: tumor stage, type of chemotherapy, number of prescribed RT fractions, before CRT hemoglobin (Hgb) levels, and body mass index (BMI). Information for each variable is summarized in Table 1. Depression was assessed by the clinician-rated 17-item Hamilton Rating Scale for Depression (HAM-D_17_) [19], using the conventional Structured Interview Guide for the HAM-D [17, 20]. The forms were administered prior, and at the last week of CRT treatment. Hamilton [19] provides detailed information on the HAM-D_17_ scale. Overall acceptable psychometric properties have been reported for the HAM-D, including internal consistency [21]. In our study, the Cronbach alphas for the HAM-D_17_ was 0.86 at before treatment and 0.84 at the end of CRT. A cutoff score of ≥15 has been used previously in cancer patients to screen for depression, with higher scores indicating more symptoms of depression [17, 22].

Total DNA was isolated from 250 mg stool aliquots collected at the end of CRT using the PowerSoil DNA Isolation Kit (MoBio, Carlsbad, CA), per the manufacturer's protocol. The V3-V4 regions of the 16S rRNA gene were amplified and sequenced using Illumina's 16S rRNA gene library preparation and sequencing workflow. Reads were trimmed at a *Q* = 25 threshold using Trim Galore! v0.44, denoised into amplicon sequence variants (ASVs) using the DADA2 plugin in QIIME2-2019.17, and the resulting ASVs were taxonomically assigned against the SILVA v132 database using a naïve Bayes classifier [16]. The ASV table was rarefied to 4,226 sequences per sample and used for alpha and beta diversity calculations in QIIME2. MetaCyc [23] pathway abundances were predicted from the rarefied table using QIIME2's q2-picrust2 plugin [24].

### 2.3. Statistical Analyses

Stool sample collection and 16S rRNA gene analysis procedures have been described previously by our group [2, 17]. Statistical differences between depression scores were computed using a two-tailed *t*-test with a significance threshold of 0.05. Associations between depressive symptom scores and microbial diversity variables were assessed using the Spearman correlation test on samples collected at the end of CRT. To fit a multiple linear regression model that adjusted for important confounding variables (e.g., age), Spearman correlations were also calculated between depressive symptom scores and taxa and pathway abundances for end of CRT samples. Automatic variable selection was performed on genera and pathways whose abundances were statistically significantly correlated with depressive symptom scores using the regsubsets function in the leap package in R. The subset of variables producing the highest *R*^2^ was used to build the final permutation-based multiple linear regression model using the lmp function in the lmPerm package in R. A *p* value cutoff of 0.05 is used for all statistical tests.

## 3. Results

### 3.1. Patient Characteristics and Depressive Symptom Scores

Study participants (*n* = 40) had a mean age of 58.9 years ± 11.3, and 58% were males (Table 2). Participants showed significantly higher mean depressive symptom scores (12.4 ± 8.7) indicating higher severity of depression at the end than before CRT (9.4 ± 7.7, *t*(47) = −3.1, *p* < 0.003). Ten participants (25%) before CRT and fifteen participants (38%) at the end of CRT had depressive symptom scores that exceeded the cutoff score for depression of ≥15 [17, 22].

### 3.2. The Gut Microbiome Is Associated with Depressive Symptom Scores at the End of CRT

In this cross-sectional pilot study, we observed no significant associations between alpha diversity, phylum abundances, and depressive symptom scores at the end of CRT (data not shown). At the genus level, however, we found that the relative abundances of Firmicutes genera, including *Gemella*, *Bacillales Family XI*, *Streptococcus*, *Lactococcus*, *Weissella*, and *Leuconostocaceae*, as well as *Actinomyces* (phylum Actinobacteria), were positively correlated with depressive symptom scores (Spearman's rho = 0.32 to 0.42). On the other hand, negative correlations between depressive symptom scores and the abundances of *Coprobacter* (phylum Actinobacteria), *Parasutterella* (phylum Proteobacteria), and other Firmicutes genera *Intestinibacter*, *Intestimonas*, *Lachnospiraceae NK4A136 group*, *Phascolarctobacterium, Ruminiclostridium*, *Ruminococcaceae (UCG-005 and uncultured)*, and *Tyzzerella* were observed (Spearman's rho = −0.43 to − 0.31) (Table 3).

**Figure 1:**
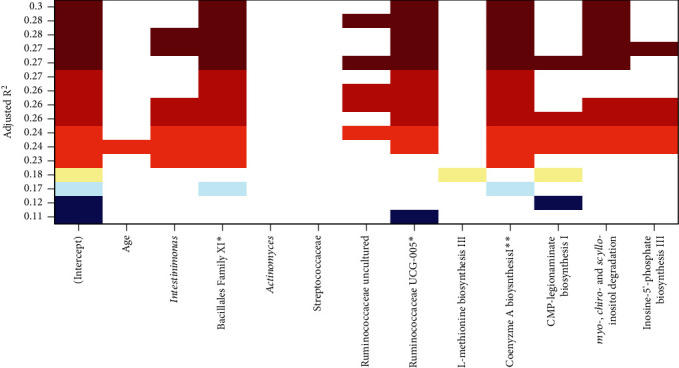
All subset regression results showing, for each subset size, the two subsets of predictor variables yielding the highest adjusted *R*^2^ for predicting depressive symptom scores. ∗ indicates predictors (coefficients) significantly correlated with depression scores, based on permutation test *p* values in the final multiple regression model.

From the ASV abundance data, 14 predicted MetaCyc [23] pathways were significantly correlated with depressive symptom scores at the end of CRT (Table 4). Eleven (~79%) of these were biosynthetic pathways, of which six (i.e., biosynthesis of inosine-5′-phosphate, L-methionine (pathways I and III), mono-*trans*, poly-*cis* decaprenyl phosphate, L-alanine, and S-adenosyl-L-methionine) were positively correlated with depressive symptoms, while five (i.e., biosynthesis of CMP-legionaminate, flavin, GDP-mannose, phosphopantothenate, and coenzyme A) were negatively correlated with depressive symptom scores (Table 4). The remaining three nonbiosynthetic pathways were negatively correlated with depressive symptom scores and included acetyl-CoA fermentation to butanoate, chondroitin sulfate degradation, and *myo*-, *chiro*-, and *scyllo*-inositol degradation pathways (Table 4). Further, with respect to multiple regression, automatic subset selection bacterial taxa measured at the end of RT and predicted pathway abundance variables showed that *Bacillales Family XI* and *Ruminococcaceae UCG-005 general* along with the predicted abundances of coenzyme A biosynthesis and *myo*-, *chiro*-, and *scyllo*-inositol degradation pathways were optimally negatively and positively correlated with depressive symptom scores, after controlling for age (Figure 1; *R*^2^ = 0.37; adjusted *R*^2^ = 0.28; SE of estimate = 7.16; *F* (_5,34_) = 3.98, *p* = 0.006).

## 4. Discussion

In this pilot study, we aimed to explore whether depressive symptom scores changed during CRT to treat rectal cancer, and if gut microbial taxa abundances and predicted functional pathways correlate with depressive symptoms at the end of CRT. While depressive symptoms are common in the general population worldwide [25], depression in the cancer population is a major health concern because it is often associated with poor adherence to treatment, higher tumor recurrence, and lower survival rates [6, 8, 26]. Our study showed that rectal cancer patients may present with increased levels of depressive symptoms even before treatment. This finding is important given that previous reports have also shown that depression is frequently underdiagnosed and undertreated [22, 27]. The physical and psychological strain associated with a patient's cancer diagnosis may be interpreted as a normal response to cancer and/or its treatment, and in the absence of a standard of care that supports ongoing clinical assessment for depression, failure to identify symptoms of depression may occur. Reasons for underdiagnosis and undertreatment of depression reported in the literature include treatment-related side effects that can mask depressive symptoms (e.g., fatigue and lack of energy), people with cancer that are expected to have poor mood, reduced time available in the treatment setting, scarce data on depression drug trials among cancer patients, and the lack of biomarkers for depression [9, 22, 27, 28]. Our findings have several clinical implications including the importance of the need for routine depression surveillance, referrals to mental health professionals, treatment of depression, and the importance of patient education before CRT initiation [2, 29], as well as the need for non-invasive objective indicator of potential depression.

Although the causal interplay between cancer, CRT, gut microbial composition, and depression remain undercharacterized, emerging evidence suggests that cancer treatments mediate changes in gut microbial composition, causing abnormalities in the gut-brain axis that may result in depressive symptoms. Briefly, one hypothesis is that changes in gut microbial composition can be affected through CRT-induced cellular responses that increase intestinal permeability and facilitate translocation of bacteria associated with peripheral inflammatory immune response [30, 31]. This increases systemic levels of proinflammatory cytokines, leading to behavioral comorbidities such as depressive symptoms [32–34]. More detailed information on the effects of cancer treatments on the gut microbiome [30, 31] and its relationship with behavioral comorbidities can be found in recent reviews [32–34]. Because the gut and central nervous systems are linked via a bidirectional communication network [35], another possibility is that the central nervous system sends signals to the gut environment, resulting in modulation of the gut microbiota composition and function [36]. For example, animal studies demonstrated that germ-free mice receiving fecal microbiota transplantation from depressed human patients presented more depressive symptoms, and their microbiota were different compared to mice receiving fecal microbiota transplantation from healthy recipients, suggesting that depressive-like symptoms can be transferred via the microbiota [37]. Conversely, probiotic intervention can alleviate and improve depressive symptoms [38, 39]. On the other hand, cancer-related symptoms, such as muscle wasting (sarcopenia; [40]), body weight loss, weakness, muscle atrophy and fat depletion (cachexia; [41–43]), anorexia [44], and social isolation [45], can also lead to depressive symptoms. In this regard, sarcopenia, cachexia, anorexia, and social isolation are also associated with microbial dysbiosis in preclinical models [41, 46–48]. For example, Pötgens et al. [41] observed an increased abundance of Enterobacteriaceae and altered gut barrier function in animal models of cancer cachexia. Taken together, the relationship between cancer, CRT, gut microbial composition, and depression is potentially complex and warrants further large-scale, controlled studies to elucidate the effects they exert on each other.

We found preliminary associations between abundances of Firmicutes, Actinobacteria, and Proteobacteria taxa and predicted pathways with depressive symptom scores at the end of CRT for rectal cancer. Firmicutes, Actinobacteria, and Proteobacteria are generally dominant in the healthy human gut [49]. However, consistent with our results, several genera from these phyla, including *Actinomyces* (phylum Actinobacteria), *Streptococcus*, and *Weissella* (phylum Firmicutes), have been associated with depression [11, 37, 50–52]. Particularly, higher *Streptococcus* abundances were detected in patients with major depressive disorders (MDD) using a similar 16S rRNA gene sequencing protocol and the same measure of depression used in our study [52]. Streptococcal infections are linked to depression-like behavior and/or to behavioral abnormalities (i.e., bacterial-infection-induced autoantibodies in pediatric autoimmune neuropsychiatric disorders associated with streptococcal infection (PANDAS)) in animal models [53, 54]. Polymicrobial infections between *Actinomyces* species and members of the *Streptococcus* genus have also been reported [55–57]. Besides infections, associations between *Actinomyces* strains and chronic inflammatory diseases such as Crohn's disease [58, 59] and higher depressive symptoms [60, 61] have also been reported. Adding to the complex association between gut microbiome and depression, there is also evidence that antimicrobials used to treat depression can result in gut dysbiosis through antimicrobial effects (Ting-Ting [62, 63]). Another Firmicutes genus that was significantly associated with depressive symptom scores, *Weissella*, was previously identified in infants born to mothers exposed to psychological stressors and distress such as intimate partner violence [64]. Nevertheless, the source of these depression-associated genera in the gut and the exact mechanisms of how they affect or are affected by brain functions and behavior require further study.

Most of the bacteria genera that significantly correlated with depressive symptoms in this study were classified to the phylum Firmicutes. Interestingly, some taxa belonging to the phylum Firmicutes (i.e., *Lachnospiraceae, Ruminiclostridium, and Ruminococcaceae*) that play roles in the short-chain fatty acid (SCFA) production were inversely associated with depressive symptoms. SCFAs (e.g., acetate, propionate, and butyrate) are microbially derived metabolites produced by the bacterial fermentation of dietary carbohydrates. SCFAs serve as a source of energy for intestinal cells, a histone deacetylase inhibitor that regulates immune homeostasis, and/or a mediator of neurotransmitter production [65]. The observed negative correlations between Lachnospiraceae and Ruminococcaceae abundances with depressive symptoms in our study were consistent with previous studies [51, 66]. Park et al. [65] proposed that a reduction in the abundances of fermentation-related bacteria may suggest a decay in SCFA production, which in turn may be associated with intestinal barrier dysfunction and an inflammatory response that can cause depression [67]. In another study, decreased levels of butyrate-producing bacteria (Ruminococcaceae_UG-013 and Lacknospiraceae_NK4A136_ group) were found to be associated with high levels of inflammatory factors such as CRP and TNF-alpha [68]. There is also clinical evidence of a positive association between stool acetate levels with depressive symptoms, as well as negative associations between butyrate and propionate levels with depressive symptoms [69]. While the associations between gut bacterial abundances, SCFA levels, gut physiology, and depressive symptoms require further study, SCFA levels have been proposed as potential noninvasive biomarkers for various health conditions [70] including depression [71]. For instance, there is clinical evidence of improvement of depression and an increase in the SCFA-producing bacteria (e.g., *Roseburia*, *Ruminococcus*, and *Eubacterium*) with an almond-based low carbohydrate diet [72] in patients with chronic diseases. Additionally, a recent review on correlations between gut bacterial metabolites with depression suggests that gut bacteria (i.e., Escherichia spp and Lactobacillus plantarum) may be a new target for depression because of their ability to regulate the neurotransmitter serotonin (5-HT) levels in the body [71]. This is important because a disrupted serotonergic system may lead to depressive symptoms [71]. As such, besides bacterial abundances, SCFAs may be useful as potential biomarkers for the assessment of depression [71] and as potential therapeutic anti-inflammatory agents [73, 74] for depression [75].

The positive correlation of *Lactobacillus* abundance and negative correlation of *Phascolarctobacterium*, *Intestimonas/Megamonas*, and *Parasutterella* abundances with depressive symptom scores in our study differed from findings from previous research. Lower *Lactobacillus* abundances were previously detected in chronic variable stress-induced depression rat model compared to controls using 16S rRNA gene sequencing [76]. Higher *Phascolarctobacterium*, *Intestimonas/Megamonas*, and *Parasutterella* abundances were previously detected in active-MDD patients compared to controls using reverse transcription-quantitative polymerase chain reaction and 16S rRNA gene pyrosequencing approaches [51]. Nevertheless, besides depression, *Streptococcus* and *Parasutterella* abundances may also be affected by rectal cancer itself or by treatment side effects [17, 73, 77]. For example, a combination of fecal bacterial candidates, including *Streptococcus bovis/Streptococcus gallolyticus*, was proposed as biomarkers for early detection of adenomatous polyps and colon cancer [73, 77]. Further, increased abundance of *Parasutterella* was related to chemotherapy-induced gastrointestinal toxicity in animal models [78]. It is, of course, possible that our study findings differed from findings in previous research because most of the available literature is based on the clinical diagnostic classification for depression while our study focuses on depressive symptoms.

Our investigation revealed the finding that our multiple regression model explained 28% of the observed variance with *p* < 0.001 and suggested that patients with higher depression scores tend to have higher levels of *Bacillales* and lower levels of *Ruminococcaceae*. Future larger-scale studies using animal models and/or anaerobic culture will be useful in confirming the functional roles of these specific taxa in depression and their utility as microbial biomarkers for depression [11]. While for our sample, bacterial genera were significantly associated with depressive symptom scores; it is important to acknowledge that depressed mood is a multifactorial condition influenced by multiple contributing factors (e.g., tumor type, prior psychological factors (preexisting mental health problems and their severity), psychological response to diagnoses (coping behavior), and social and contextual factors (unemployment and lack of social support); [79]. However, findings of our study suggest that gut microbiome disruption may be present before overt clinical depression is recognized, a finding that may prove useful in future research aimed at identifying biomarkers to aid in the establishment of clinical depression. Associations between other relevant factors including social and contextual factors potentially contributing to depressive symptoms among people with cancer have been supported in prior exhaustive reviews [79]. Further research that includes biological factors, as well as behavioral factors such as lack of coping strategy for handling the trauma of a cancer diagnosis, is required. This knowledge may shed light on personalized medicine/interventions for risk factor reduction.

As previously stated, the underpinnings of CRT-related depressive symptoms have not been fully elucidated. Based on the MetaCyc pathway predictions [80], depressive symptoms appeared to correlate mainly with biosynthetic pathways (e.g., flavin biosynthesis and S-adenosyl-L-methionine biosynthesis). This is remarkable since our pathway predictions point to mechanisms that researchers have proposed to contribute to the biologic underpinnings of depression: gut dysbiosis resulting in low production of micronutrients and gut dysbiosis promotion of inflammation [11, 80]. In fact, riboflavin (vitamin B2) deficiency has been associated with depression in women [81], and the flavin biosynthesis I pathway was inversely associated with depressive symptoms in our study. Acetyl CoA fermentation to butanoate II was also inversely associated with depressive symptoms. This is interesting because there is evidence that acetyl-CoA is one of the pathways known for the production of the anti-inflammatory SCFA butyrate [17, 80, 82]. These findings also have further research implications since riboflavin supplementation has been suggested to improve mood in individuals [83], and sodium butyrate administration was reported to have antidepressant-like effects in animal models [84]. However, others have found in animal models that administration of the sodium propionate (also a SCFA) but not sodium butyrate induces antidepressant-like effects [85]. Further, the finding that the S-adenosyl methionine biosynthesis pathway was negatively correlated with depressive symptom scores is interesting since S-adenosyl methionine has been used as an antidepression supplement [86]. Two degradation pathways (chondroitin sulfate and *myo*-, *chiro*-, and *scyllo*-inositol) were also negatively correlated with depressive symptom scores. Chondroitin sulfate has been found to play an important role in synaptic transmission and plasticity in animal models [87] while evidence suggests that inositol may play a role in the management/alleviation of depression [88]. Conversely, the positive correlation of mono-trans, poly-cis decaprenyl phosphate biosynthetic pathway abundances with depressive symptom scores may be linked to similar increases in Actinobacteria (such as Actinomyces) abundances with depressive symptom scores, since this pathway is found in Mycobacterium species classified to the phylum Actinobacteria [89, 90]. Therefore, these candidate pathways and their metabolites warrant further investigation as they may shed light on the assessment and treatment of CRT-related symptoms such as depression and fatigue [14].

## 5. Limitations

Our results must be interpreted with caution as our pilot study has notable limitations including the brief duration of the follow-up and lack of a control group. For example, we cannot rule out that the influence of CRT on depressive symptoms could be due to the cancer nor can we rule out the confounding effects of cancer and CRT on the gut microbiome composition. Inclusion of a control group of patients without CRT could have strengthened the inference on the influence of CRT on depressive symptoms, as would the collection of stool samples before CRT to assess microbiota changes impacted by the CRT administration and controlling for CRT side effects. Additional studies with larger numbers of participants are needed to further examine the role of other factors (e.g., tumor samples, lifestyle, social environmental factors, pain, stress, diet, and diarrhea) that may affect the variability of the gut microbiota measurements and/or depressive symptoms in patients with rectal cancer. As the outcomes of this study were dependent on clinician-rated depressive symptoms, future research may also consider comparing our findings with participants' self-reported depressive symptoms perhaps using other validated measures of depression (e.g., the PROMIS depression forms). The availability of a larger sample size would permit the exclusion of somatic symptoms of depression in future studies. Controlling for these and other behavioral manifestations of depressive symptoms may inform the impact of these symptoms on gut microbiota and address a needed gap in the literature.

## 6. Conclusions

Overall, our results indicated that rectal cancer patients experience increases in depressive symptoms before CRT, which can worsen after treatment. We also identified taxa and predicted pathways that were significantly correlated with depressive symptom scores. Our findings served to inform future larger-scale clinical studies involving not only genus-level 16S rRNA gene analysis but also metatranscriptomics, metagenomics, culturing experiments, and animal models. These future studies will contribute to the body of the literature of the underpinnings of depressive symptoms and clinical depression and facilitate the identification of microbial targets for effective diagnosis and clinical interventions.

## Figures and Tables

**Table 1:** Summary of measurements.

Variables	Method of measurement	Frequency
Depressive symptoms	17-item Hamilton Rating Scale for Depression (HAM-D_17_)	Before and end of treatment
Gut microbiome	Stool samples for 16S rRNA sequencing	End of treatment
Age, gender, occupational status	Demographic form	Before treatment
Type of chemotherapy, tumor stage, number of treatments, body mass index, hemoglobin	Health form information gathered from their health record	Before treatment

**Table 2:** Study participants' (*n* = 40) demographic and clinical characteristics.

Characteristics	Participants (*n* = 40)
Gender	
M	23 (58%)
Occupation	
Working	17 (42%)
Retired	13 (33%)
Handicapped	10 (25%)
Chemotherapy	
5FU	17 (42%)
Xeloda	23 (58%)
	Median (IQR)
Age	58 (53, 67)
Tumor stage	3 (3, 3)
Number of treatments	28 (23, 33)
BMI	27.5 (23.6, 30.1)
Hemoglobin	11.8 (10.7, 13.2)

BMI = body mass index.

**Table 3:** Statistically significant correlations between genus relative abundances and the Hamilton Depression Rating Scale scores at the end of CRT.

Genus	End-CRT	
	Rho	*p* value
Phylum: Actinobacteria		
*Actinomyces*	0.40	0.01
*Actinomycetaceae*	0.40	0.01
*Actinomycetales*	0.38	0.02
Phylum: Bacteroidetes		
*Coprobacter*	-0.32	0.05
Phylum: Firmicutes		
*Bacillales Family XI*	0.42	0.007
*Gemella*	0.42	0.007
*Intestinibacter*	-0.37	0.02
*Intestinimonas*	-0.43	0.005
*Lachnospiraceae NK4A136 group*	-0.32	0.05
*Lactococcus*	0.34	0.03
*Phascolarctobacterium*	-0.32	0.04
*Ruminiclostridium 6*	-0.39	0.01
*Ruminococcaceae UCG-005*	-0.32	0.05
*Ruminococcaceae uncultured*	-0.41	0.009
*Streptococcaceae*	0.37	0.02
*Streptococcus*	0.37	0.02
*Tyzzerella*	-0.35	0.03
*Weissella*	0.32	0.04
Phylum: Proteobacteria		
*Parasutterella*	-0.31	0.05

CRT = chemotherapy and radiation therapy.

**Table 4:** Statistically significant correlations between MetaCyc pathway abundances and the Hamilton Depression Rating Scale scores at the end-CRT samples.

MetaCyc pathway	End-CRT	
	Rho	*p* value
Acetyl-CoA fermentation to butanoate II	-0.37	0.02
Chondroitin sulfate degradation I (bacterial)	-0.33	0.04
CMP-legionaminate biosynthesis I	-0.41	0.009
Flavin biosynthesis I (bacteria and plants)	-0.36	0.02
Inosine-5′-phosphate biosynthesis III	0.33	0.04
GDP-mannose biosynthesis	-0.33	0.04
L-methionine biosynthesis I	0.36	0.02
L-methionine biosynthesis III	0.42	0.007
Mono-*trans*, poly-*cis* decaprenyl phosphate biosynthesis	0.36	0.02
*Myo*-, *chiro*-, and *scyllo*-inositol degradation	-0.39	0.01
Phosphopantothenate biosynthesis I	-0.39	0.02
Superpathway of coenzyme A biosynthesis I (bacteria)	-0.42	0.007
Superpathway of L-alanine biosynthesis	0.36	0.02
Superpathway of S-adenosyl-L-methionine biosynthesis	0.33	0.04

CRT = chemotherapy and radiation therapy.

## Data Availability

The data used to support the findings of this study are available from the corresponding author upon request. It will be deposited in a database when the study is complete.
